# Long-Term Effects of a Ketogenic Diet for Cancer

**DOI:** 10.3390/nu15102334

**Published:** 2023-05-16

**Authors:** Ryuichiro Egashira, Michiko Matsunaga, Akimitsu Miyake, Sayaka Hotta, Naoko Nagai, Chise Yamaguchi, Mariko Takeuchi, Misaki Moriguchi, Satoko Tonari, Mai Nakano, Hitomi Saito, Keisuke Hagihara

**Affiliations:** 1 Department of Advanced Hybrid Medicine, Graduate School of Medicine, Osaka University, Osaka 565-0871, Japan; r_egashira@kanpou.med.osaka-u.ac.jp (R.E.); paprika3c5@gmail.com (M.M.); s_hotta@kanpou.med.osaka-u.ac.jp (S.H.); m_takeuchi@kanpou.med.osaka-u.ac.jp (M.T.); moriguchi@kanpou.med.osaka-u.ac.jp (M.M.); tonari@kanpou.med.osaka-u.ac.jp (S.T.); norse.vsta.m@gmail.com (M.N.); h_saito@kanpou.med.osaka-u.ac.jp (H.S.); 2 Japan Society for the Promotion of Science, Tokyo 102-0083, Japan; 3 Department of AI and Innovative Medicine, Tohoku University School of Medicine, Sendai 980-8575, Japan; akimitsu.miyake.d5@tohoku.ac.jp; 4 Division of Nutritional Management, Osaka University Hospital, Osaka 565-0871, Japan; nagaink@hosp.med.osaka-u.ac.jp (N.N.); ychise@hosp.med.osaka-u.ac.jp (C.Y.)

**Keywords:** cancer, ketogenic diet, long-term effect, propensity score weighting

## Abstract

A ketogenic diet has been proposed as a potential supportive therapy for cancer patients, although its long-term influence on survival rates remain controversial. In our previous report, we presented promising results for 37 of 55 patients with advanced cancer enrolled between 2013 and 2018 who remained on a ketogenic diet for at least 3 months. We followed all 55 patients until March 2023 and analyzed the data up to March 2022. For the 37 patients with previously reported promising results, the median follow-up period was 25 (range of 3–104) months and 28 patients died. The median overall survival (OS) in this subset of 37 patients was 25.1 months and the 5-year survival rate was 23.9%. We also evaluated the association between the duration of the ketogenic diet and outcome in all 55 patients, except for 2 patients with insufficient data. The patients were divided into two groups: those who followed the diet for ≥12 months (*n* = 21) and those who followed it for <12 months (*n* = 32). The median duration of the ketogenic diet was 37 (range of 12–99) months for the ≥12 months group and 3 (range of 0–11) months for the <12 months group. During the follow-up period, 41 patients died (10/21 in the ≥12 months group and 31/32 in the <12 months group). The median OS was 19.9 months (55.1 months in the ≥12 months group and 12 months in the <12 months group). Following the inverse probability of treatment weighting to align the background factors of the two groups and make them comparable, the adjusted log-rank test showed a significantly better OS rate in the group that continued the ketogenic diet for a longer period (*p* < 0.001, adjusted log-rank test). These results indicate that a longer continuation of the ketogenic diet improved the prognosis of advanced cancer patients.

## 1. Introduction

In recent years, the importance of proper nutrition in supporting the treatment of patients with malignant tumors has been emphasized [1]. A ketogenic diet, a high-fat and low-carbohydrate diet, is a long-standing dietary therapy for central nervous system diseases, e.g., refractory seizure syndrome [2]. In recent years, the usefulness of ketogenic diets for malignant tumors has been investigated, and animal studies using various mice models have shown that ketogenic diets improve survival outcomes [3,4]. In humans, randomized, controlled trials in ovarian and endometrial cancer showed improvement in the physical component of quality of life [5], selectively reduced fat mass, and significantly reduced blood insulin levels [6]. However, studies of survival outcome have been controversial. Although there have been reports of improved overall survival (OS) [7], the validity of the results has been debated because there were concerns about the study design or methodology [8,9]. The degree of carbohydrate restriction (approximately 20 g to 50 g in RCTs) and the duration of ketogenic diets (9 days to 3 months in RCTs) in previous studies are inconsistent, and it is difficult to generalize the data obtained in these studies [10]. In addition, the duration of ketogenic diets and follow-up periods in many studies have been as short as a few months. Overall, although there is some evidence that ketogenic diets may be beneficial in the treatment of malignant tumors, more research is needed to determine the optimal approach to using this dietary therapy.

We developed an original regimen of ketogenic diets for cancer patients as follows: carbohydrates were restricted to 10 g/day during week one, 20 g/day from week two for three months, and 30 g/day three months later. A total of 55 patients with advanced cancer were enrolled from February 2013 to December 2018 and followed up by November 2019. We reported the results of 37 evaluable cases who consumed a ketogenic diet for more than 3 months. Median OS was 32.2 (maximum: 80.1) months, and the three-year survival rate was 44.5% [11]. These results implied that a longer duration of the ketogenic diet would likely have a greater impact on the outcomes of advanced cancer patients. However, the duration required to be effective is not clear, and the duration of the ketogenic diet varied widely from patient to patient in previous reports. We followed all patients who have taken up the ketogenic diet for cancer and confirmed the outcomes of all patients up to March 2023. We then analyzed the follow-up data from these 55 patients to determine whether there was an effect of the duration of the ketogenic diet on the OS of advanced cancer patients.

In this report, the long-term effects of a ketogenic diet for cancer in the 37 cases reported from the previous study is presented; the median OS was 25.1 months, and the five-year survival rate was 23.9%. The ketogenic diet-ABC (KD-ABC) score composed of Alb, BS, and CRP values, which were found to be related to prognosis in a previous report, was also confirmed. To examine the effect of the continuation of the ketogenic diet, the outcomes were compared between the group that continued the ketogenic diet for more than one year with those of the group that did not. A propensity score-weighting method was used to align the background factors of the two groups and make them comparable. The group that continued the ketogenic diet for more than 12 months showed a significantly better OS than the group that did not.

## 2. Materials and Methods

### 2.1. Participants and Design

A single-center, case-series study was conducted at Osaka University Hospital to investigate the effect of the ketogenic diet on patients with advanced cancer. The patients were enrolled from February 2013 to December 2018. The previous study included patients who were diagnosed with stage IV cancer by histology or cytology, had a performance status ≤2, and could take foods orally. Patients who could not ingest foods orally, had a performance status >3, or had diabetes mellitus were excluded from the study. Finally, 37 patients were evaluable after remaining on the ketogenic diet for at least 3 months and undergoing regular check-ups. The primary endpoint was tumor size measured by positron emission tomography–computed tomography (PET-CT) three months after starting the ketogenic diet. The secondary endpoints were clinical responses one year after starting the ketogenic diet. The ketogenic diet was continued according to the patients’ wishes and their health condition. Details of the implementation protocol are available in the previously published report that reported outcomes as of November 2019 [11].

### 2.2. Ketogenic Diet for Cancer Patients

The ketogenic diet was administered under the guidance of a registered dietitian at our hospital. The details of our ketogenic diet regimen were reported previously [11]. Briefly, the most stringent carbohydrate restriction (<10 g/day) was used during the first week. From the second week, for three months, the carbohydrate restriction was 20 g/day. After three months, for patients who wished to continue the ketogenic diet, the carbohydrate restriction was 30 g/day or less (≤10 g/one meal). Patients’ outcomes were monitored on an on-going basis while they were on the ketogenic diet or after the ketogenic diet was completed. Until March 2023, all patients were followed up. Telephone or mail contact was primarily used to determine if the patient was alive, and if death had occurred, the date of death was obtained. Demographic information, primary tumor information, treatment history, blood test results, body composition, and EORTC-QLQ30, collected in previous studies at induction and at 3 months, were collected retrospectively and used in the present analysis.

### 2.3. Equipment and Evaluation Tools

Blood samples were tested at the central clinical laboratory of Osaka University. The InBody 720 (Biospace Co., Ltd., Seoul, Republic of Korea) was used to measure body composition. In a previous report, the patients’ quality of life was assessed using the EORTC-QLQ-C30 questionnaire, which was developed and validated by the European Organization for Research and Treatment of Cancer [12]. The questionnaire includes 30 questions, which were converted into 15 assessment items using the methodology developed by the EORTC Quality of Life Study Group.

### 2.4. Statistics

#### 2.4.1. Long-Term Outcomes and Stratification of Survival Curves

Survival curves to March 2022 for the 37 previously reported cases were plotted using the Kaplan–Meier method. OS was defined as the time from obtaining informed consent to last follow-up or until all-cause death. Patients who were alive at last follow-up were treated as censored cases. Survival curves for colorectal and lung cancers, for which there were a particularly large number of cases, were plotted in the same way.

In a previous report, we reported survival curve stratification based on albumin, blood glucose, CRP at three months, and the KD-ABC (Ketogenic Diet Albumin Blood Glucose CRP) score, taking these variables into account [11]. In the present study, the same analysis was performed, looking at long-term outcomes.

#### 2.4.2. Survival Analysis Using Propensity Score Weighting

To determine whether a prolonged ketogenic diet increases survival time, the patients were divided into two groups and compared: those who remained on the ketogenic diet for more than 12 months and those who remained on the diet for less than 12 months. In such a retrospective, non-randomized study, the standard Kaplan–Meier estimate may be biased due to confounding factors. IPTW survival analysis is one approach to reduce the influence of confounding factors [13]. This method consists of two main steps. First, the probability of being exposed given the individual patient’s covariates, which is the so-called propensity score, is calculated using a multivariate logistic regression model. Second, the weighting of each individual patient by the inverse of the propensity score is then performed, so that those with a higher propensity score are assigned a lower weight and those with a lower propensity score are assigned a higher weight. The assignment of these weights to the study population generates the “pseudo-population” in which the imbalance of a set of observed covariates is expected to become balanced between two groups.

In the context of the present research, the propensity score represents the probability of whether each patient stays on the ketogenic diet for 12 months or longer, conditional on a set of empirical evidence-based covariates. The covariates were selected based on the assumption that the factors contributing to long-term persistence on a ketogenic diet would consist of two components: factors that contribute to long-term survival and psychosocial factors that support the continuation of the diet. Balance in covariates between two groups before and after IPTW was assessed using the standardized mean difference (SMD). Cumulative incidence estimates before and after IPTW were calculated using the usual Kaplan–Meier estimates and the IPTW-adjusted Kaplan–Meier estimates, respectively. The standard log-rank test and adjusted log-rank test with IPTW were used to compare the survival distributions between two groups. Two-sided *p*-values lower than 0.05 were considered significant.

All analyses were performed by independent biostatisticians using R software version 4.2.1 (R Project for Statistical Computing). The R packages ‘survival’, and ‘survey’ were used to obtain survival curves and before and after IPTW-adjusted Kaplan–Meier estimates, respectively.

### 2.5. Ethics

This study was approved by the genome committee of Osaka University (Approval number 526). Written consent was obtained from all patients.

## 3. Results

### 3.1. Patient Characteristics

**Figure 1:**
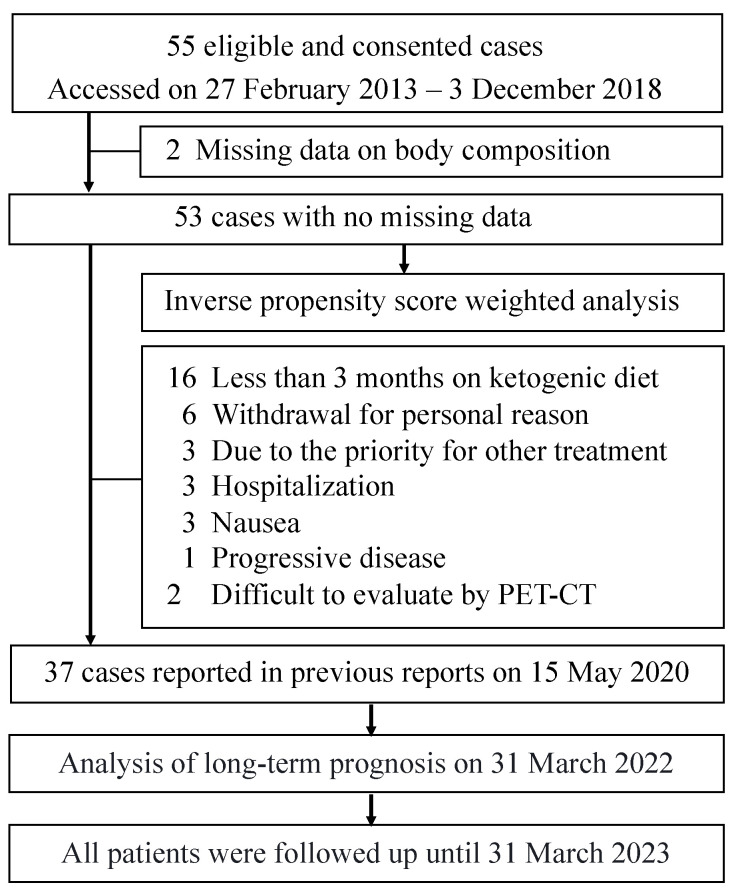
Flowchart of patient selection for the analysis.

In the previous report, 55 patients met the entry criteria and were enrolled in the trial. Of these, 37 patients who had been on a ketogenic diet for at least three months and had imaging results to assess malignant tumor progression were included in the analysis. All 55 cases were followed for outcome. Two patients dropped out due to deteriorating physical condition immediately after consent was obtained, and data on their body composition were missing. Therefore, only 53 patients were available for analysis (Figure 1).

The descriptive statistics (mean ± standard deviation or median and interquartile range) of the patients are shown in Table 1. The mean age at the beginning of the study was 55.5 ± 12.1 years. When the follow-up period was defined as the date of last known survival or the date of death, the patients had a median follow-up period of 19 months (44 in the ≥12 months group (*n* = 21) and 11.5 in the <12 months group (*n* = 32)), a minimum follow-up period of 56 days, and a maximum follow-up period of 104 months. The median duration of the ketogenic diet was 37 (range of 12–99) months for the ≥12 months group and 3 (range of 0–11) months for the <12 months group. During the follow-up period, 41 patients died (10/21 in the ≥12 months group and 31/32 in the <12 months group), and the median OS was 19.9 months (55.1 months in the ≥12 months group and 12 months in the <12 months group).

### 3.2. Long-Term Prognosis and Stratification of Survival Curves

**Figure 2:**
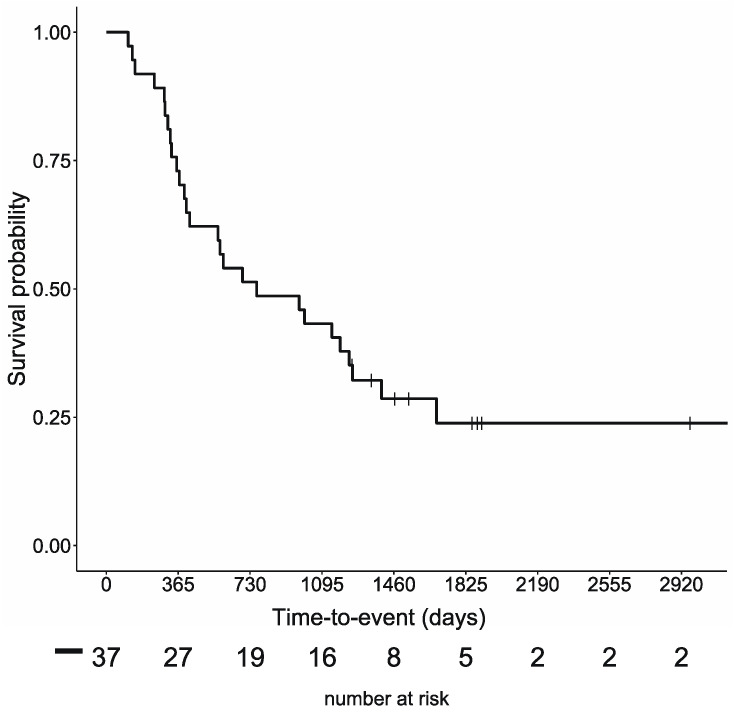
Overall survival rates for the 37 cases.

The survival curves for the previously reported 37 cases are shown in Figure 2. For these 37 cases, the mean age at the beginning of the study was 54.8 ± 12.6 years, and the median follow-up period was 25 (range of 3–104) months. Twenty-eight patients died during the follow-up period, with a median OS of 25.1 months and a 5-year survival rate of 23.9%. For patients with colorectal cancer, eight patients remained on the ketogenic diet for more than three months. The mean age at the beginning was 49.3 ± 13.7 years, and the median follow-up period was 19 (10–62) months. The median duration of the ketogenic diet was 11.5 (4–48) months. Six patients died during the follow-up period, and the median OS was 19 months (Figure S1). For patients with lung cancer, six patients remained on the ketogenic diet for more than three months. The mean age at the beginning was 56.7 ± 5.5 years, and the median follow-up period was 41.5 (9–104) months. The median duration of the ketogenic diet was 16.5 (3–99) months. Four patients died during the follow-up period, and the median OS was 33.1 months (Figure S2).

**Figure 3:**
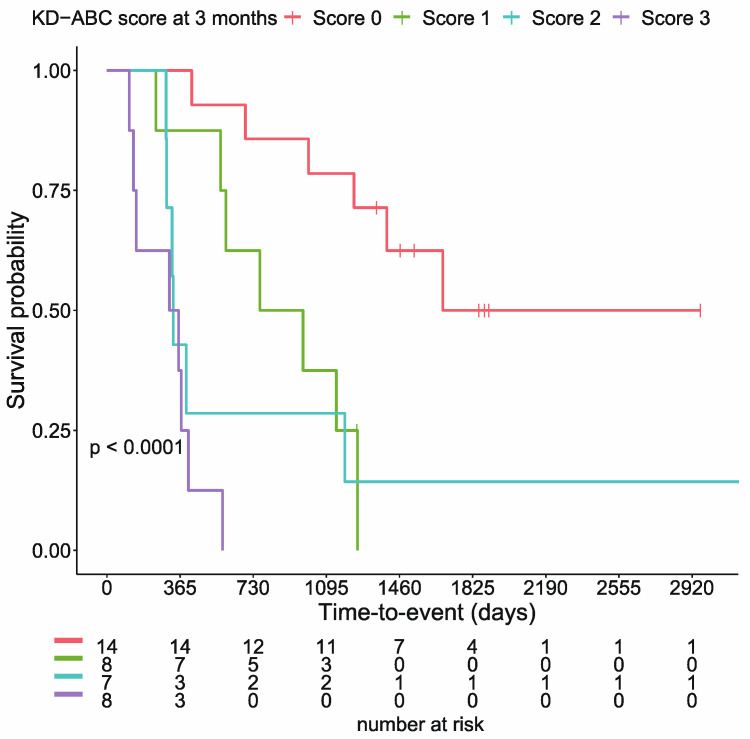
KD-ABC scores significantly stratify cumulative survival rates (*p* < 0.0001, log-rank test).

In a previous report, we showed that Alb, CRP, blood glucose, and the KD-ABC score (calculated from these three variables) separated the 37 cases into groups with significantly different survival rates. In the present study, as in previous reports, Alb, CRP, and blood glucose levels at three months separated the survival curves into groups with significantly different survival rates in cancer patients (Figure S3). Similar results were obtained for the KD-ABC score composed of Alb, BS, and CRP values (*p* < 0.0001, log-rank test; Figure 3).

### 3.3. Effects of the Duration of the Ketogenic Diet on Survival

**Figure 4:**
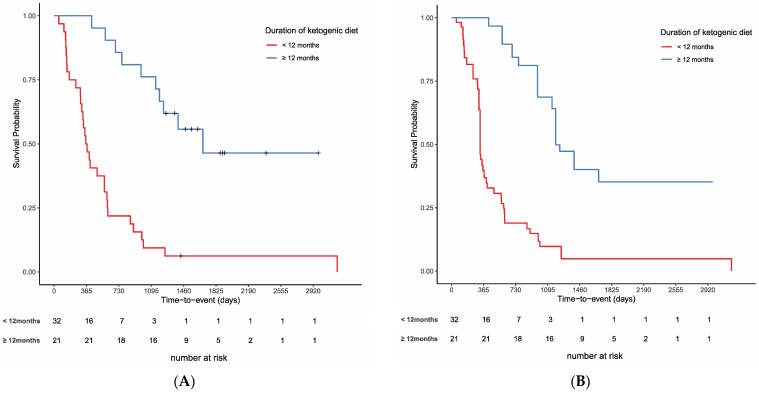
Overall survival rates for the 37 cases. (**A**) Unadjusted comparison of patients who had been on a ketone diet for more than 12 months and those who had not (*p* < 0.001, log-rank test). (**B**) Comparison of the two groups on the inverse propensity score-weighted analysis (*p* < 0.001, adjusted log-rank test).

Figure 4A shows survival curves based on the Kaplan–Meier method for the two groups of patients on the ketogenic diet for more than 12 months and less than 12 months. Before weighting, the survival rate in the ≥12 months group was significantly higher than that of the <12 months group (log-rank test, *p* < 0.001). To examine the effects of the duration of the ketogenic diet on survival, IPTW analysis was performed with the white blood cell count (WBC), creatinine, alkaline phosphatase (ALP), neutrophil count, C-reactive protein (CRP), and albumin as factors contributing to survival in the propensity score from the blood tests. Lung cancer was included as a factor for worse prognosis. Global health status, physical functioning, role functioning, social functioning, pain, and appetite loss were included from EORTC-QLQ30 as factors contributing to survival. To the best of our knowledge, there is no evidence on factors contributing to longer persistence on a ketogenic diet in cancer patients. Social functioning, which assesses whether treatment interferes with social life, and constipation, which is often seen as an adverse event, were considered likely to influence long-term persistence with the ketogenic diet for cancers. Although performance status (PS) is a clinically important prognostic factor, PS was not used, because all patients in the present study were in relatively good general condition at enrollment.

Table 2 shows the results of the comparison of the two groups after IPTW using propensity scores. Before weighting, there were 32 cases in the <12 months group and 21 cases in the ≥12 months group, and after adjustment, there were 54.2 cases in the <12 months group and 41.8 cases in the ≥12 months group. IPTW succeeded in balancing the selected covariates to some extent across the cohort, as evidenced by the improved SMDs. Prior to IPTW implementation, many SMDs were above 0.4. Propensity score-matched adjustment reduced the SMDs to near or below 0.3 for all 14 variables. The distribution of propensity scores before and after the adjustment is shown in Figure S4. The adjusted difference between two groups decreased after weighting, but the adjusted log-rank test showed that the OS rate was significantly better in the group that continued the ketogenic diet for more than 12 months (*p* < 0.001, adjusted log-rank test; Figure 4B).

### 3.4. List of Long-Term Survivors on a Ketogenic Diet for Cancer

Of the 37 cases, those alive for 5 years at the end of March 2023 are listed in Table 3, and a brief report on patient number 8 follows.

**Figure 5:**
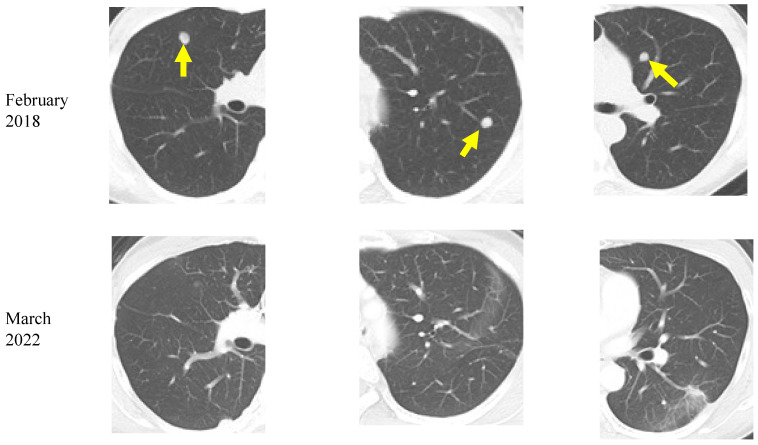
Chest computed tomography images of a colorectal cancer patient with lung metastases (yellow arrows). A decrease in bilateral multiple lung metastases 4 years after the start of the ketogenic diet is seen.

A 48-year-old woman was diagnosed with RAS-mutant colon cancer and multiple bilateral lung metastases in December 2016. After laparoscopic low anterior resection, she was treated with six cycles of Xelox (capecitabine and oxaliplatin) and two cycles of Xeloda (capecitabine) as adjuvant chemotherapy. However, a CT in February 2018 showed the appearance of new lung metastatic lesions. In April 2018, the patient was referred to our institution, and she started a ketogenic diet at age 50. She also continued standard chemotherapy at the other institution. Between January and March 2019, radiofrequency ablation (RFA) was performed at the other institution for bilateral lung metastases. Liver metastases were found in June, and transarterial embolization (TAE) and RFA were performed in July. A CT in December showed reduced lung and liver metastases. During the above treatment, the patient continued a ketogenic diet. Chest and abdominal CT in March 2022 showed no increase in liver or lung metastatic lesions (Figure 5). As of March 2023, the patient was 55 years old, continues on a ketogenic diet, and was in good general condition. During treatment, potassium chloride and statins were administered orally for mild hypokalemia and hyperlipidemia, respectively, but no serious side effects were observed.

## 4. Discussion

In this report, the long-term effects (up to 10 years) of a ketogenic diet on outcomes in advanced cancer in 55 cases at our department in Osaka University Hospital were examined. Patients who were able to continue the ketogenic diet for at least 12 months were compared with those who were not, using IPTW with propensity scores. The results show that patients who were able to continue the ketogenic diet for a longer period had significantly better outcomes than those who were not.

In a previous case series study, patients could be stratified into groups with significantly different survival rates using CRP, albumin, blood glucose, and the KD-ABC score 3 months after the beginning of the ketogenic diet. In the current analysis, taking long-term outcomes into account, patients were clearly shown to be divided into groups with significantly different outcomes based on albumin (≥ or <4.0 mg/dL), blood glucose (> or ≤90 mg/dL), CRP (> or ≤0.5 mg/dL), and the KD-ABC score (0–3). CRP and albumin are important factors that define the prognosis of malignancy [1,14], and this was reaffirmed. Although blood glucose is not often mentioned as a prognostic factor in malignancies, a significant increase in progression-free survival (PFS) has been reported when comparing patients on a ketogenic diet with median blood glucose levels below 83.5 mg/dL on day 6 to those above the median [15]. Some argue that blood glucose stability is more important than the blood ketone body levels [16], and more clinical studies are needed.

The results for lung cancer and colorectal cancer were shown separately, although their numbers were very small. As of March 2023, at least 4 years and 5 months had passed since consent was obtained for patients with Stage IV lung cancer or colorectal cancer who had been on a ketogenic diet for at least 3 months. Two lung cancer patients were alive with an actual measured survival rate of 33.3%. The measured 5-year survival rate for Stage IV lung adenocarcinoma cancer patients diagnosed between 2003 and 2014 in Japan was 8.0% [17].

Two colorectal cancer patients were alive, with an actual measured survival rate of 25.0%. The case of a patient with RAS-mutant colon cancer with lung and liver metastases, who started the ketogenic diet approximately 16 months after diagnosis because the metastases were not controlled despite standard treatment, was described. Although RFA for lung metastases is not a standard treatment, it is performed at some centers mainly for patients who do not respond to continued standard treatment [18]. The measured 5-year survival rate for colorectal cancer was 19.8% in Japan, and the 5-year relative survival rate for metastatic colorectal cancer was 14%, according to the report from the American Cancer Society [19], so it is worth noting that the patient has survived 5 years (as of March 2023) in good general condition with no metastatic growth.

The efficacy of a ketogenic diet in malignant tumors has been frequently reported in animal studies [3], but clinical studies in humans are insufficient [10]. Several prospective, single-arm trials have also examined survival and response rates. Few randomized trials have performed sample size calculations with survival or response rates as primary endpoints [7,20].

Khodabakhshi et al. reported a significant improvement in OS in post-operative breast cancer patients on a ketogenic diet for 90 days [7]. Voss et al. reported a 9-day program incorporating a ketogenic diet and intermittent calorie restriction for recurrent brain tumors, with no significant effects on 6-month PFS [15,20]. Van der Louw et al. compared the results with a historical cohort and found no prognostic improvement [21]. Rieger et al. compared PFS with a control group selected from within the same center and reported comparable rates [22], and two other studies presented only descriptive statistics on survival [23,24]. Both studies had small numbers of cases (between 8 and 20) and differed in terms of cancer type and treatment, making it difficult to integrate and interpret the results. A retrospective study with 359 colorectal cancer cases reported a borderline significant reduction in cancer-specific survival in the subgroup with a blood glucose load of less than 100 g/day and fat as a percentage of calorie intake of 40% or more [25]. These results indicate that the evidence on the survival and related outcomes of a cancer ketogenic diet is quite insufficient.

Possible reasons for this can include inadequate carbohydrate restriction, short duration of implementation, and extreme limitation of protein in cancer patients. Khodabakhshi et al. reported a relatively long-term intervention of 90 days with carbohydrate restriction to 6% of energy and protein intake to 19%, whereas Voss et al. restricted them to 50 g per day, with many other important differences. On the other hand, our ketogenic diet regimen has no calorie restriction, with a clear target value and period for carbohydrate restriction. Based on our results and the above considerations, we believe our ketogenic diet regimen is promising for cancer patients.

In the present study, the long-term efficacy of the ketogenic diet was evaluated using propensity scores to make it possible to compare between groups. We hypothesized that the factors contributing to long-term persistence on a ketogenic diet consist of those factors contributing to long-term survival and factors supporting the continuation of the diet. Since the number of patients was not large, the variables were selected mainly with reference to clinical evidence and considering the degree of variability in both groups. There is no consensus set of the survival-predictive indices that can be applied to all patients with any given type of cancer in relatively good general condition, and we used a variety of clinical studies to determine the most important ones. There is evidence that WBC, ALP [26], creatinine, neutrophils, CRP, and albumin [27] contribute to survival. The number of lung cancer patients was disproportionate in the two groups, and lung cancer is also associated with poor outcomes [28]. Global health status [29], physical functioning [30], role functioning [29], social functioning [31], pain [32], and appetite loss [33] in the EORTC-QLQ30 are known to contribute to survival. Social functioning is considered to be a psychosocial factor that supports the continuation of diet therapy, and appetite loss is considered to prevent the continuation of a diet that is not familiar to the patient. Constipation is a highly prevalent adverse event on the ketogenic diet and is likely to interfere with the continuation of the diet [21]. By adjusting for these factors, we considered that a certain degree of comparability was ensured.

There are several limitations to the present study. First, this was a retrospective study, and it was not possible to adjust for unmeasured factors. Therefore, the possibility that the lack of adjustment by propensity score may have affected the results cannot be ruled out. Second, a variety of cancer types, their histologic types, and genetic backgrounds are included. The efficacy of a ketogenic diet for malignant tumors is naturally expected to vary depending on these background factors, and future studies should take these factors into account.

## 5. Conclusions

The long-term outcomes of patients with advanced cancer who were treated with a ketogenic diet at our department in Osaka University Hospital were reported. A propensity score-weighted analysis showed that a ketogenic diet continued for more than 12 months may significantly improve OS. We emphasize that well-designed, prospective, controlled trials with sufficient power are needed to reach a conclusion on this issue.

## Figures and Tables

**Table 1:** Patient characteristics.

	<12 Months Group	≥12 Months Group	Total
Number of cases, *n*	32	21	53
Age, y	57.6 ± 12.8	52.2 ± 10.0	55.5 ± 12.1
Sex, male/female, *n*	14/18	8/13	22/31
Primary cancer, *n*			
Non-small cell lung cancer, *n*	3	11	14
Colorectal cancer, *n*	4	5	9
Breast cancer, *n*	4	2	6
Pancreatic cancer, *n*	1	3	4
Head and neck cancer, *n*	3	1	4
Bone and soft tissue sarcoma, *n*	3	1	4
Ovarian and peritoneal cancer, *n*	1	2	3
Biliary tract cancer, *n*	0	2	2
Endometrial cancer, *n*	0	1	1
Bladder cancer, *n*	0	2	2
Brain tumor, *n*	1	0	1
Esophageal cancer, *n*	0	1	1
Gastric cancer, *n*	0	1	1
Prostate cancer, *n*	1	0	1
Follow-up time, median (range), months	11.5 (1–104)	44 (13–97)	19 (1–104)
Duration of KD, median (range), months	3 (0–11)	37 (12–99)	6 (0–99)

Abbreviations: KD, ketogenic diet; OS, overall survival time.

**Table 2:** Clinical characteristics before and after IPTW.

	Unadjusted				Adjusted			
	<12 Months (*n* = 32)	≥12 Months (*n* = 21)	*p*-Value	SMD	<12 Months (*n* = 54.2)	≥12 Months (*n* = 41.8)	*p*-Value	SMD
Male (%)	14 (43.8)	8 (38.1)	0.902	0.115	20.0 (36.9)	20.0 (47.9)	0.526	0.223
Age, y	57.62 (13.01)	52.24 (10.24)	0.116	0.46	53.28 (15.86)	54.38 (9.84)	0.839	0.083
Weight, kg	54.02 (12.85)	55.28 (11.32)	0.716	0.104	57.02 (16.28)	55.87 (11.36)	0.819	0.082
BMI	20.38 (3.42)	20.88 (3.26)	0.604	0.147	21.59 (4.20)	20.83 (2.99)	0.553	0.207
Lean mass of limb, kg	16.94 (4.50)	16.87 (4.16)	0.955	0.016	17.06 (4.75)	17.45 (4.47)	0.808	0.084
Lung cancer, *n* (%)	11 (34.4)	3 (14.3)	0.192	0.482	13.8 (25.5)	10.2 (24.4)	0.945	0.025
Laboratory tests								
Platelets, 10^4^/μL	23.65 (9.09)	22.44 (4.83)	0.579	0.166	21.49 (8.62)	20.84 (5.61)	0.793	0.09
WBC, 10^3^/μL	5.75 (2.57)	4.53 (1.87)	0.066	0.545	5.07 (2.45)	4.65 (1.93)	0.58	0.191
Creatinine, mg/dL	0.74 (0.22)	0.67 (0.19)	0.269	0.318	0.71 (0.20)	0.74 (0.23)	0.762	0.111
ALP, U/L	366.50 (432.68)	224.38 (85.62)	0.145	0.456	307.15 (344.13)	236.01 (83.23)	0.207	0.284
Potassium, mEq/L	4.07 (0.37)	4.10 (0.26)	0.674	0.123	4.01 (0.35)	4.05 (0.31)	0.768	0.108
BUN, mg/dL	16.75 (5.62)	16.29 (4.86)	0.758	0.088	16.26 (4.75)	17.71 (5.84)	0.449	0.272
Neutrophil count, 10^3^/μL	3.86 (1.99)	2.67 (1.39)	0.022	0.69	3.22 (1.95)	2.84 (1.37)	0.528	0.222
CRP, mg/dL	1.11 (1.69)	0.20 (0.37)	0.02	0.741	0.72 (1.41)	0.36 (0.56)	0.253	0.33
Albumin, g/dL	3.88 (0.41)	4.21 (0.33)	0.003	0.894	4.04 (0.41)	4.16 (0.32)	0.362	0.31
EORTC-QLQ30								
Global health status	51.30 (24.04)	62.70 (25.50)	0.106	0.46	58.01 (24.75)	60.25 (26.88)	0.813	0.087
Financial difficulties	29.16 (33.60)	22.21 (26.52)	0.429	0.229	22.35 (30.63)	25.02 (24.52)	0.74	0.096
Physical functioning	82.29 (15.10)	88.58 (13.19)	0.126	0.444	85.66 (14.51)	86.76 (12.24)	0.804	0.082
Role functioning	75.00 (25.75)	85.71 (26.50)	0.149	0.41	80.79 (24.32)	81.29 (28.78)	0.959	0.019
Emotional functioning	77.08 (20.52)	79.37 (14.10)	0.658	0.13	79.87 (18.23)	76.65 (18.47)	0.64	0.175
Cognitive functioning	83.85 (17.70)	81.74 (24.09)	0.716	0.1	83.20 (16.00)	72.76 (33.33)	0.368	0.399
Social functioning	69.27 (29.97)	82.55 (19.34)	0.078	0.527	72.94 (26.62)	78.37 (18.71)	0.399	0.236
Fatigue	35.05 (19.38)	36.49 (21.99)	0.803	0.07	28.45 (19.14)	42.56 (28.25)	0.165	0.584
Pain	23.45 (21.93)	11.91 (18.37)	0.052	0.57	17.36 (20.09)	11.31 (19.80)	0.358	0.304
Dyspnea	15.61 (16.88)	15.86 (17.04)	0.959	0.015	14.44 (16.77)	18.64 (16.94)	0.465	0.249
Insomnia	30.20 (30.95)	28.57 (36.95)	0.863	0.048	30.55 (26.59)	36.88 (42.64)	0.631	0.178
Nausea and vomiting	7.82 (16.39)	7.94 (12.49)	0.977	0.008	5.20 (13.55)	8.45 (12.12)	0.377	0.253
Appetite loss	30.20 (28.54)	20.63 (28.82)	0.24	0.334	27.98 (25.66)	27.38 (37.48)	0.963	0.019
Constipation	16.66 (25.40)	28.56 (30.34)	0.129	0.425	18.40 (23.55)	22.03 (26.87)	0.628	0.144
Diarrhea	13.54 (26.59)	11.10 (24.34)	0.737	0.096	8.72 (22.33)	11.49 (24.91)	0.686	0.117

Data presented as means (standard deviation). Abbreviations: ALP, alkaline phosphatase; BMI, body mass index; BUN, blood urea nitrogen; CRP, C-reactive protein; EORTC-QLQ, European Organization for Research and Treatment of Cancer Quality of Life Questionnaire; WBC, white blood cell count.

**Table 3:** Patients alive for 5 years or more.

No	Sex	Age (y) *	Cancer Type	Histology	TNM Classification	Continuation of KD	Duration of KD, Weeks	OS (Months)	Current Status ^†^
1	F	56	Lung cancer	Adenocarcinoma	T2aN0M1a	finished	16	121	deceased
2	F	52	Lung cancer	Adenocarcinoma	T2aN0M1b	Ongoing	422	109	alive
3	F	36	Colorectal cancer	Tubular adenocarcinoma	T4bN1aM1b	finished	53	74	alive
4	F	41	Breast cancer	Invasive ductal carcinoma	T3N1M1	finished	61	73	alive
5	M	79	Sarcoma	Chondrosarcoma	T2N1M1	Ongoing	263	72	alive
6	M	46	Oral and pharyngeal cancer	Adenoid cystic carcinoma	T4aN1M1	finished	121	81	deceased
7	M	55	Lung cancer	Adenocarcinoma	T3N1M1a	Ongoing	218	62	alive
8	F	50	Colorectal cancer	Tubular adenocarcinoma	TXN1bM1a	Ongoing	207	60	alive

* Age at introduction of ketogenic diet; † Status at end of March 2023. Abbreviations: KD, ketogenic diet; OS, overall survival.

## Data Availability

Not applicable.

## Supplementary Materials

The following supporting information can be downloaded at: https://www.mdpi.com/article/10.3390/nu15102334/s1. Figure S1: Overall survival rates for 8 cases of colorectal cancer; Figure S2: Overall survival rates for 6 cases of non-small cell lung cancer; Figure S3: Stratified overall survival rates for 37 cases. (A) Patients with serum values for albumin (Alb) ≥ or <4.0 mg/dL (*p* < 0.0001). (B) Glucose (Glu) > or ≤90 mg/dL (*p* < 0.0001) and (C) C-reactive protein (CRP) > or ≤0.5 mg/dL (*p* < 0.0001); Figure S4: Distributions of propensity scores before and after adjustment.
