# Supplementary material for: Long-Term Effects of a Ketogenic Diet for Cancer
Source: Nutrients. 2023 May 16;15(10):2334. doi: 10.3390/nu15102334 (PMC10221628; doi:10.3390/nu15102334)
Supplement: Supplementary file 1 [file nutrients-15-02334-s001.zip › nutrients-2342952-supplementary.pdf]

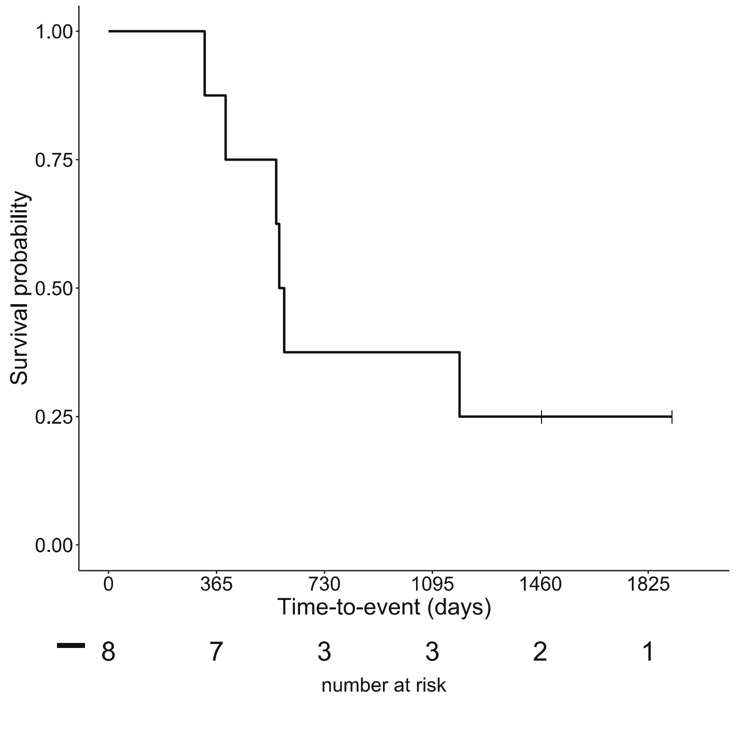

Figure S1. Overall survival rates for 8 cases of colorectal cancer.

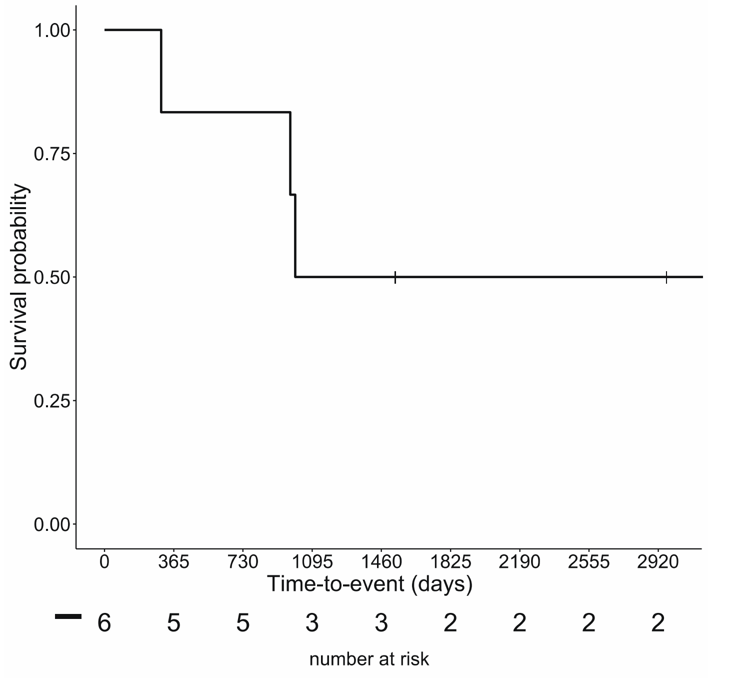

Figure S2. Overall survival rates for 6 cases of non-small cell lung cancer.

(A)

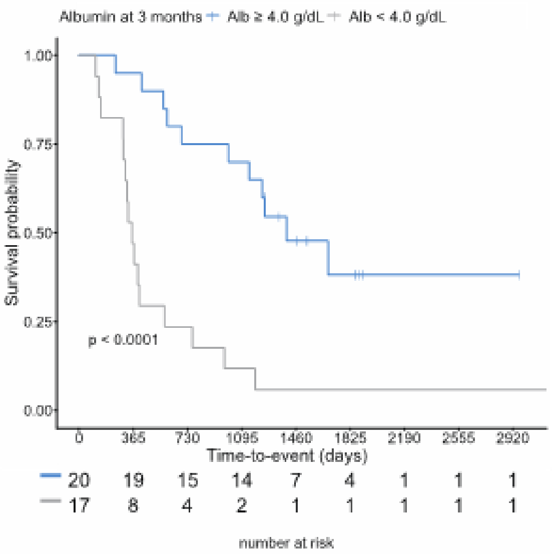

Albumin at 3 months + Alb ≥ 4.0 g/dL + Alb < 4.0 g/dL
Survival probability
1.00
0.75
0.50
0.25
0.00
p < 0.0001
0 365 730 1095 1460 1825 2190 2555 2920
Time-to-event (days)
20 19 15 14 7 4 1 1 1
17 8 4 2 1 1 1 1 1
number at risk

(B)

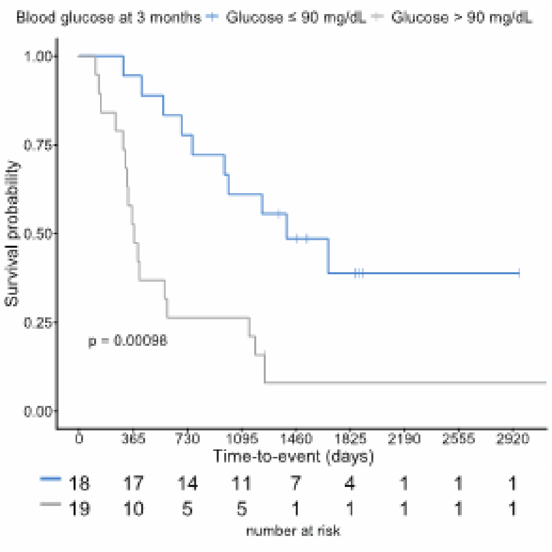

Blood glucose at 3 months + Glucose ≤ 90 mg/dL + Glucose > 90 mg/dL
Survival probability
1.00
0.75
0.50
0.25
0.00
p = 0.00098
0 365 730 1095 1460 1825 2190 2555 2920
Time-to-event (days)
18 17 14 11 7 4 1 1 1
19 10 5 5 1 1 1 1 1
number at risk

(C)

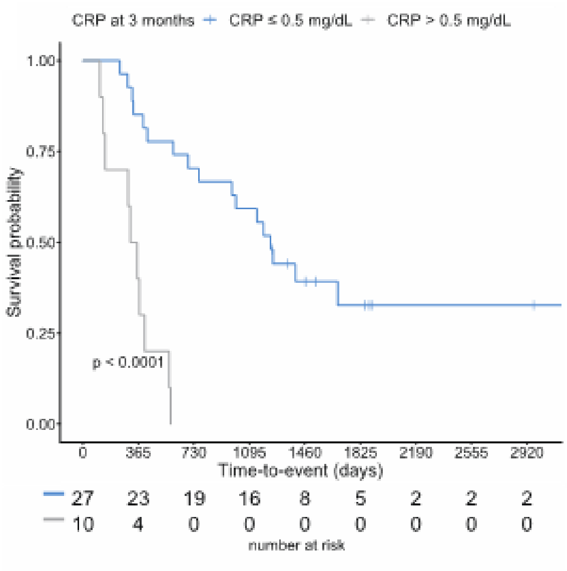

Figure S3. Stratified overall survival rates for 37 cases. (A) Patients with. serum values for albumin (Alb) ≥ or < 4.0 mg/dL ($p < 0.0001$). (B) glucose (Glu) > or ≤ 90 mg/dL ($p < 0.0001$) and (C) C-reactive protein (CRP) > or ≤ 0.5 mg/dL ($p < 0.0001$).

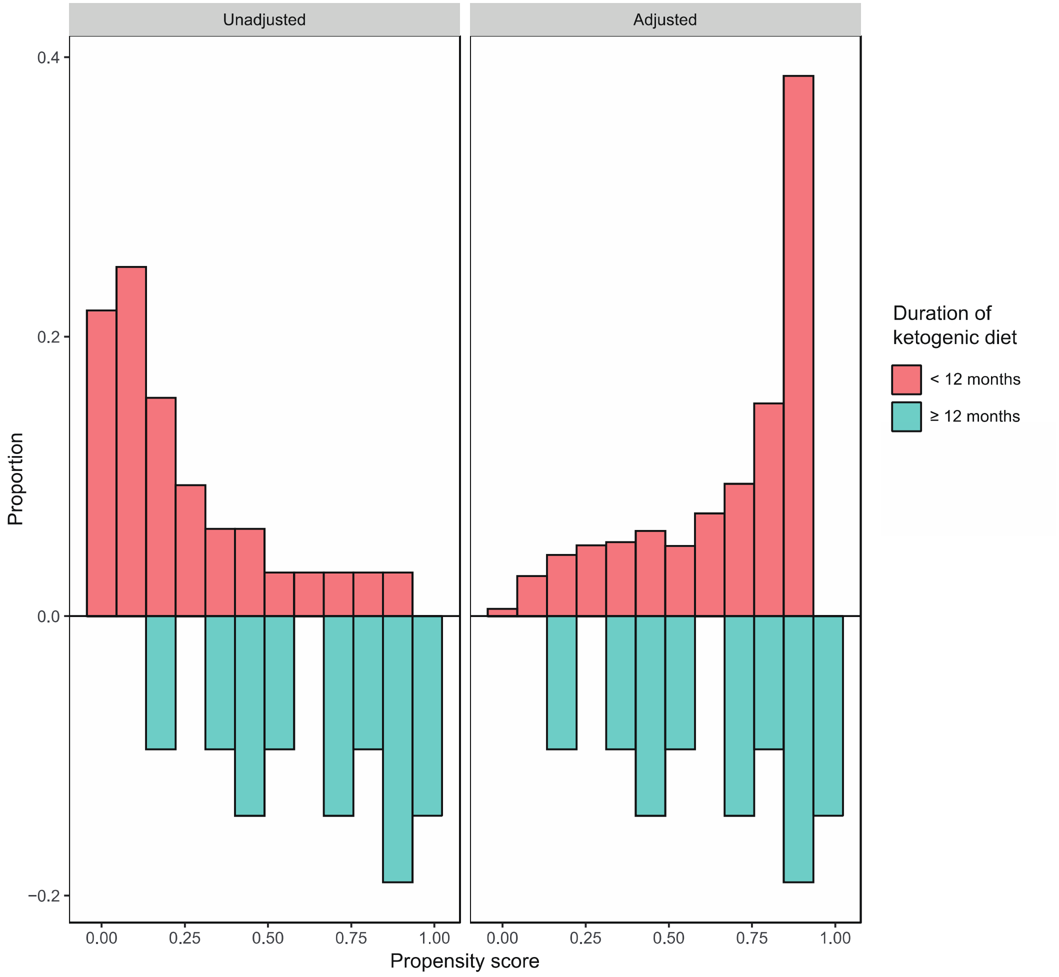

Figure S4. Distributions of propensity scores before and after adjustment.
